# Patient-Oriented Digital Health Intervention for Chronic Kidney Disease: From Evidence to Everyday Care

**DOI:** 10.2196/108605

**Published:** 2026-08-26

**Authors:** Maoliosa Donald, Dwight Sparkes, Matthew Thomas James

**Affiliations:** 1 Department of Medicine and Community Health Sciences, University of Calgary, 3280 Hospital Drive, Calgary, AB, T2N4Z6, Canada, 1 403-220-2465, 1 403-210-6660

**Keywords:** chronic kidney disease, self-management, digital health

## Abstract

Lightfoot and colleagues explored patient perspectives on the implementation and sustainability of My Kidneys & Me, a digital self-management intervention for chronic kidney disease, identifying key factors that may influence successful adoption into routine care. The study shows that generating evidence of effectiveness alone is insufficient for successful mobilization into practice, highlighting the importance of embedding implementation evaluation in concert with clinical trials to identify practical strategies that support real-world uptake and sustained use.

In health care, self-management refers to the daily behaviors and lifestyle choices that allow people to live well with a chronic condition. It is especially relevant for people with early-stage chronic kidney disease (CKD) because their actions, together with medical interventions, can help slow progression to kidney failure and reduce cardiovascular complications, thereby improving longevity, quality of life, and well-being. However, people with early-stage CKD may not prioritize self-management because of limited awareness and inadequate support, which can reduce their ability, motivation, and willingness to participate actively and effectively in care that prevents CKD-related complications. Improving patient awareness and knowledge is therefore a central component of self-management interventions aimed at improving health outcomes. In a recent article published in the *Journal of Medical Internet Research*, Lightfoot et al [1] report findings from a qualitative study exploring how people with CKD perceived the implementation and long-term sustainability of My Kidneys & Me, a digital health intervention co-developed in the United Kingdom to provide specialist health and lifestyle education that supports self-management for people living with CKD and their families.

Self-management supports need to consider the systematic provision of care and resources that enable patient activation (the degree to which an individual understands their role in the care process and possesses the knowledge, skill, and confidence to manage their own health and health care) [2,3]. Digital health tools are well suited to this task because they can deliver continuous, personalized, and scalable support that complements episodic health care encounters [4,5]. CKD requires patients to make daily decisions about medications, lifestyle, symptom monitoring, and when to seek care—tasks that occur largely outside of clinical settings. Digital interventions can provide timely education, behavioral support, reminders, goal setting, symptom tracking, and information at the time of need, empowering individuals to manage their condition more effectively between health care visits. My Kidneys & Me previously demonstrated efficacy in improving patient activation among people with CKD stage 3 or 4 and low activation at baseline in the SMILE-K (Self-Management Intervention Through Lifestyle Education for Kidney Health) randomized trial [6].

In the current study*,* Lightfoot et al [1] used a reflexive thematic analysis of 42 semistructured interviews involving 35 participants from the SMILE-K trial and identified five interrelated themes influencing successful implementation: (1) delivering information at the right time and tailored to individual needs; (2) promoting the program through multiple communication channels; (3) ensuring equitable access by addressing barriers such as digital literacy and digital poverty; (4) building trust through endorsement by health care professionals; and (5) integrating the intervention into routine clinical pathways to support sustained engagement. These findings illustrate how successful translation of digital health interventions into routine CKD care will depend on more than demonstrating clinical effectiveness; implementation strategies need to be patient-centered, flexible, and responsive to the needs of individuals. Participants emphasized that readiness to engage with digital resources varies across the disease trajectory, underscoring the need for individualized timing rather than a standardized approach, using integrated approaches with the care system and supported by trusted care providers. By identifying practical barriers and facilitators from the patient perspective, this study provides important knowledge that can now be used to inform an implementation framework that promotes equitable access, long-term engagement, and sustainable uptake into care.

Importantly, this work represents a critical step forward in a coherent program of research that began with the co-design of the My Kidneys & Me intervention [7] alongside patients, care providers, and researchers and progressed through evaluation in the SMILE-K randomized controlled trial, demonstrating effectiveness while simultaneously exploring implementation and sustainability from the patient perspective. This blended approach exemplifies an increasingly important model for clinical research, in which implementation considerations are embedded alongside traditional effectiveness evaluation rather than deferred until after efficacy has been established [8,9]. Effectiveness-implementation hybrid designs enable simultaneous evaluation of intervention effectiveness and implementation determinants, facilitating earlier identification of barriers and facilitators, accelerating translation into practice, and generating evidence that is more relevant to health system decision-making.

Lightfoot and colleagues [1] have identified important challenges to be anticipated with broad implementation of My Kidneys & Me, providing a foundation to design effective implementation strategies that will support uptake and sustainability within daily, routine kidney care. Frameworks such as exploration, preparation, implementation, sustainment (EPIS) can now be applied to these findings to address not only factors that are relevant to context but also bridging factors that acknowledge the interrelated nature of inner and outer settings, as well as innovation factors that reflect the characteristics of the intervention [10]. Figure 1 illustrates how the 4 phases of EPIS could build upon the knowledge from this study to guide the implementation process.

**Figure 1.**
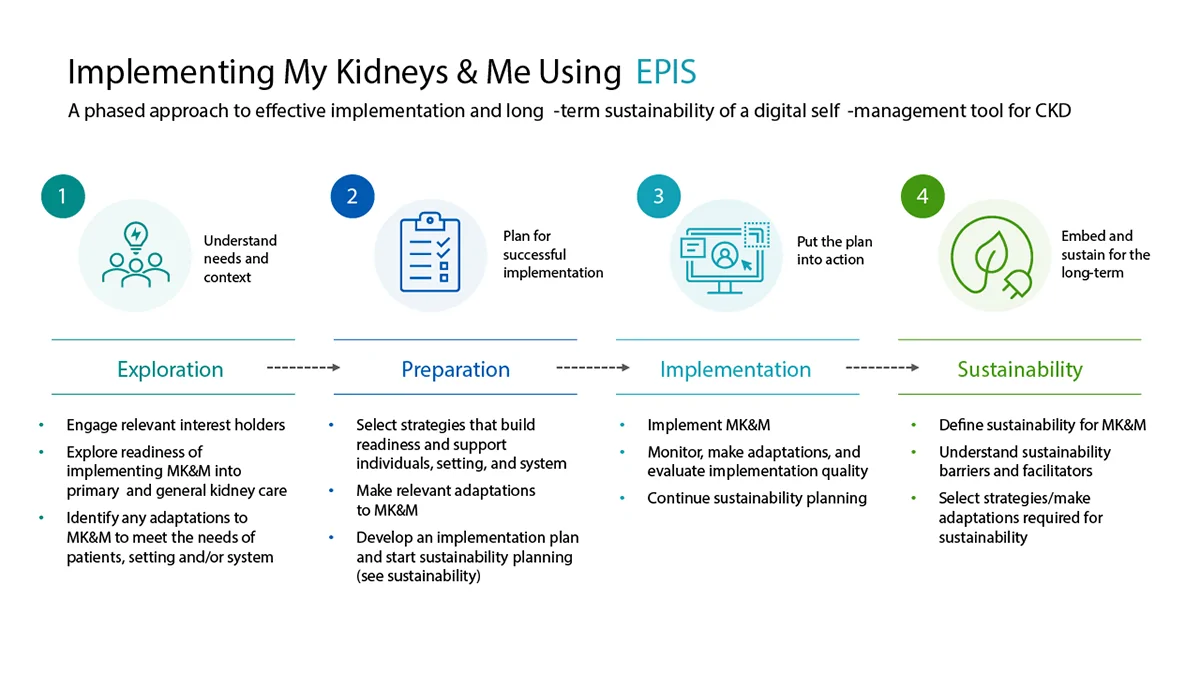
Implementing My Kidneys & Me (MK&M) Using the EPIS (exploration, preparation, implementation, sustainment) framework. CKD: chronic kidney disease.

By proactively identifying contextual factors that shape adoption, integration, and sustained use, Lightfoot et al [1] provide actionable evidence to support real-world implementation and help narrow the gap between research and practice in CKD self-management. This integrated research continuum offers a model for other chronic disease digital health interventions, showing how co-design, effectiveness testing, and implementation evaluation can be deliberately connected to increase the likelihood of an intervention’s success and accelerate its integration into routine clinical care.

## Abbreviations

CKD: chronic kidney disease
EPIS: exploration, preparation, implementation, sustainment
SMILE-K: Self-Management Intervention Through Lifestyle Education for Kidney Health
